# Inequalities in Trends in Healthy and Disability-Free Life Expectancies: A Systematic Review

**DOI:** 10.1093/geroni/igaa057.2196

**Published:** 2020-12-16

**Authors:** Gemma Spiers, Fiona Beyer, Dawn Craig, Barbara Hanratty, Carol Jagger

**Affiliations:** 1 Newcastle University, Newcastle upon Tyne, England, United Kingdom; 2 Population Health Sciences Institute, Newcastle University, Newcastle upon Tyne, England, United Kingdom

## Abstract

To update previous reviews, we searched Medline, Embase, Scopus and the Office for National Statistics (ONS) website for studies and reports published after 2016 that describe trends in healthy life expectancy, active life expectancy or disability-free life expectancy (DFLE) in the UK and other OECD high-income countries. We focus here on studies reporting inequalities by socioeconomic position (SEP) in these trends. There was mixed evidence of educational and area-level deprivation inequalities in trends in DFLE, with four studies indicating that educational inequalities were widening in European countries. No studies were identified that examined inequalities in disability-free life expectancy trends in the UK. All studies were based on cross-sectional data from multiple time points or longitudinal panel studies. We discuss the size of inequalities in DFLE between SEP groups and the limitations of previous studies.

